# Goleman’s Leadership styles at different hierarchical levels in medical education

**DOI:** 10.1186/s12909-017-0995-z

**Published:** 2017-09-19

**Authors:** Anurag Saxena, Loni Desanghere, Kent Stobart, Keith Walker

**Affiliations:** 1 St. Andrews College, College of Medicine, University of Saskatchewan, Rm 412, 1121 College Drive, Saskatoon, SK S7N 0W3 Canada; 2 College of Medicine, University of Saskatchewan, 5D40 Health Sciences Building Box 19, 107 Wiggins Road, Saskatoon, SK S7N 5E5 Canada; 3 College of Education, University of Saskatchewan, Room 3079 28 Campus Drive, Saskatoon, SK S7N 0X1 Canada

**Keywords:** Leadership, Medical education, Leadership styles, Emotional intelligence

## Abstract

**Background:**

With current emphasis on leadership in medicine, this study explores Goleman’s leadership styles of medical education leaders at different hierarchical levels and gain insight into factors that contribute to the appropriateness of practices.

**Methods:**

Forty two leaders (28 first-level with limited formal authority, eight middle-level with wider program responsibility and six senior- level with higher organizational authority) rank ordered their preferred Goleman’s styles and provided comments. Eight additional senior leaders were interviewed in-depth. Differences in ranked styles within groups were determined by Friedman tests and Wilcoxon tests. Based upon style descriptions, confirmatory template analysis was used to identify Goleman’s styles for each interviewed participant. Content analysis was used to identify themes that affected leadership styles.

**Results:**

There were differences in the repertoire and preferred styles at different leadership levels. As a group, first-level leaders preferred democratic, middle-level used coaching while the senior leaders did not have one preferred style and used multiple styles. Women and men preferred democratic and coaching styles respectively. The varied use of styles reflected leadership conceptualizations, leader accountabilities, contextual adaptations, the situation and its evolution, leaders’ awareness of how they themselves were situated, and personal preferences and discomfort with styles. The not uncommon use of pace-setting and commanding styles by senior leaders, who were interviewed, was linked to working with physicians and delivering quickly on outcomes.

**Conclusions:**

Leaders at different levels in medical education draw from a repertoire of styles. Leadership development should incorporate learning of different leadership styles, especially at first- and mid-level positions.

**Electronic supplementary material:**

The online version of this article (10.1186/s12909-017-0995-z) contains supplementary material, which is available to authorized users.

## Background

The need to develop leadership competencies in physicians stems from the recognition that physician leaders support and drive change in reforming healthcare systems [1–4]. Leadership development in medicine is now emphasized for practicing physicians [5] as well as during their education [6, 7], and is reflected in competency-based medical education [8–12].

Ultimately, leadership development is aimed at effective leadership behaviors. Since leadership is a process of intentional influence [13–15], a leader’s behavior towards others is at the heart of leadership. As defined in the Merriam-Webster dictionary, the word “style” refers to “a way of behaving or doing things” [16]. At its core then, leadership style is the leader’s interactions with others. The success of leaders within organizations is not dependent on what they aim to do, but rather on *how* they do it. Of the many underlying factors that affect leadership behaviour, such as intentions and motivations, there has been considerable importance attached to emotional intelligence (EI).

EI is “the ability to monitor one’s own and others’ feelings and emotions, to discriminate among them and to use this information to guide one’s thinking and actions” [17] (p188). EI is generally conceptualized as having four overarching domains - self-awareness, self-management, social awareness, and relationship management - embracing eighteen different competencies [18]. EI has been linked to better interpersonal relations [19] and compassionate and empathetic patient care, and better communication and professionalism skills [20]. Despite concerns with the reliability and validity of EI measures [21], EI has been linked to effective leadership in many professional arenas [18, 22–24], including in medicine [20, 25, 26], hence a model of leadership styles based upon EI. Additionally, it has been incorporated as a key aspect of learning the leader role in the CanMEDS 2015 tools guide [27].

Goleman’s work on leadership styles incorporates EI [28] and is based on the studies carried out by the Hay Group (as referenced in [29]), which claimed that EI accounts for more than 85% of exceptional performance in top leaders. These leadership styles are understood in terms of the leaders’ underlying EI capabilities and each style’s causal link with outcomes [28]. The most effective leaders act according to one or more of six distinct leadership styles depending on the situation: visionary (syn. Authoritative – outlining the vision and allowing innovations and experimentation), coaching (developing long-term goals based upon peoples’ strengths and weaknesses), affiliative (promoting harmony and personal relationships), democratic (emphasizing teamwork and collaboration), pacesetting (focusing on learning new approaches and performance to meet challenging goals), and commanding (seeking immediate compliance) [18]. Although successful leaders are able to adapt the type of leadership style they use to a specific situation or circumstance [30], many leaders may use one style more often than others, which compromises their effectiveness.

Given EI’s link to interpersonal behaviors and leadership effectiveness, Goleman’s six leadership styles are useful for investigating leadership behaviors in medical education. The purpose of this study was to identify Goleman’s leadership styles used by medical education leaders, to delineate any differences across participant groups (first-, middle- and senior-level leaders; *study phase I*) and to lend insight into the factors that contribute to the appropriateness of the practices in different leadership roles (*study phase II*). The findings are likely to have implications for individual practice, leadership development and recruitment of future leaders.

## Methods

### Participants

The participants were medical education leaders at various levels at the College of Medicine of the University of Saskatchewan and at senior-level nationally in Canada. Based upon Adair’s work [31], participants were grouped into one of three formal hierarchical leadership levels: First-level (with limited formal responsibility e.g., medical student and resident leaders), middle-level (responsibilities for larger cross-discipline programs such as undergraduate curriculum and postgraduate programs e.g., course coordinators, curriculum chairs, program directors), and senior-level (with higher and wider responsibility e.g., Associate Deans and Deans). In phase I of this study, participants were recruited from all hierarchical leadership levels within the College of Medicine. Phase II involved only senior-level leaders (who did not participate in phase I of this study) with either a provincial mandate or leadership positions in national level educational organizations (see Table 1).

**Table 1:** Displays the demographic information for first- middle- and senior- level leaders

Category	Total number of participants	Gender (M:F ratio)	Age range (mean)	Years in medical education leadership position(s) (mean)	Leadership positions
Event Study					
First-level	28	4:3	23–29 (25)	3–5 (3)	Chief Residents, Undergraduate Student Leaders
Middle-level	8	7:1	37–64 (52)	8–19 (13)	Program, Course Coordinators, Curriculum Chairs, Directors of Academic Centres
Senior-level	6	3:3	49–68 (57)	8–24 (18)	Associate & Assistant Deans and Dean
Semi-structured interviews					
Senior-level	8	5:3	48–68 (57)	10–20 (15.7)	Associate Deans, Senior leaders in national level medical education organizations

**Table 2:** Displays the leadership styles identified by first- middle- and senior- level leaders as being most frequently used in their practice (top three rankings)

Rank	Leadership styles	First-level leaders	Middle-level leaders	Senior leaders
1	Visionary	25%	25%	33%
	Coaching	11%	50%	17%
	Affiliative	11%	0%	33%
	Democratic	50%	25%	17%
	Pacesetting	4%	0%	0%
	Commanding	0%	0%	0%
2	Visionary	14%	25%	17%
	Coaching	43%	38%	17%
	Affiliative	11%	13%	0%
	Democratic	21%	25%	50%
	Pacesetting	11%	0%	17%
	Commanding	0%	0%	0%
3	Visionary	21%	13%	17%
	Coaching	29%	13%	50%
	Affiliative	21%	38%	17%
	Democratic	21%	25%	17%
	Pacesetting	4%	13%	0%
	Commanding	4%	0%	0%

**Table 3:** a) Displays the mean rankings for First- Middle- and Senior- leaders, as well as across gender, for the six leadership styles. Within groups, leadership styles represented with an asterisk were found to be significantly more used (p < .05) than bolded leadership styles

a) Phase I (Mean rankings of different participant groups)						
	Visionary	Coaching	Affiliative	Democratic	Pace-setting	Commanding
First level leaders	2.93	2.61	**3.61**	**1.86** *	**4.49**	**5.41**
Mid-level leaders	3.06	1.63*	3.63	2.63	**4.56**	**5.50**
Senior leaders	2.67	2.678	3.0	2.33*	4.75	**5.58**
Women (mean rank)	2.20	2.50	4.0	1.90*	**4.90**	**5.50**
Men (mean rank)	3.35	1.77*	3.15	2.69	**4.50**	**5.54**
b) Phase II (Use of leadership styles by senior leaders who were interviewed)						
Participant	Visionary (most commonly used)	Coaching	Affiliative	Democratic (most commonly used)	Pace-setting (when required)	Commanding (when required)
1	yes	x	x	yes	yes	yes
2	yes	yes	yes	yes	yes	yes
3	yes	x	x	yes	yes	yes
4	yes	x	x	yes	yes	yes
5	yes	yes	x	yes	yes	yes
6	yes	x	x	yes	yes	yes
7	yes	yes	yes	yes	yes	yes
8	yes	x	yes	yes	yes	yes

b) Provides an overview of the leadership styles Senior-level leaders identified as using in the semi-structured interviews

#### Phase I

Recruitment letters were sent to all current and six previous leaders at the College of Medicine. The response rate to participate in the study was 35% for the first level leaders, 27% for the middle level leaders and 33% for the senior level leaders. There were 28 first-level, eight middle-level, and six senior-level participants.

#### Phase II

Semi-structured interviews of eight additional senior medical education leaders (as defined above) selected through purposive sampling were conducted by researcher AS; ten senior level leaders were contacted and eight agreed to participate (response rate: 80%).

### Materials and procedure

#### Phase I

To explore differences in leadership conceptualizations between groups, participants were first instructed to provide a simple written definition of their perception of leadership. To gather data on differences in the leadership styles, the participants filled out a questionnaire, which asked them to reflect on their experiences as a leader, and rank order Goleman’s leadership *styles.* The participants ranked Goleman’s six styles from most- to least commonly used by ranking their most preferred leadership style as 1, the next preferred leadership style a 2, and so on. If a leadership style was not used, then either a “x” was put against it or left blank, this was later coded by the researchers for analysis purposes as a 7. A Brief description of each leadership style was provided to participants. Qualitative responses of the leadership definitions were thematically categorized within each group and common themes are reported. Descriptive statistics were used to explore the dominant leadership style within each participant group and between gender; for gender differences for first level leaders, results are based on available demographics (9 participants only). Friedman tests were used to determine if there were differences in ranked leadership styles within each leadership group as well as by gender. For any significant effects (*p* < 0.05), Wilcoxon signed-rank tests, with Bonferroni corrections applied for multiple comparisons, were used to determine which domains differed in their rankings.

#### Phase II

The semi-structured interview questions were framed to encourage the participants to recall stories and experiences to explore a deeper understanding of their leadership behaviors and describe their leadership styles. Additional questions that explored their interactions with stakeholders included recall and descriptions of when they led a major change and had to rally people around them (see Additional file 1). The interview questions were pilot tested to establish the trustworthiness and credibility of the questionnaire (*n* = 2) and based upon the pilot data the questions were revised to generate sharing of unique and extraordinary experiences and encourage imagination (see Additional file 1). The interviews incorporated an “ethic of care” [32] aimed at developing trust and openness between the researcher and the participant(s) by attempting to become “co-equals” conversing about a mutually relevant subject. The data were collected by note taking and tape recording. The interview transcripts and the notes were analyzed by one author (AS) in two ways. First, these were reviewed to identify Goleman’s styles for each participant based upon the descriptions of these styles and the process of confirmatory template analysis [33, 34]. Secondly, transcripts were inductively analyzed through content analysis process of coding to identify common themes that affect leadership styles [35, 36].

## Results

### Phase I

#### Definition of leadership

There were two common themes in the conceptualization of leadership by the **first-level leaders**; these included, 1) providing direction when assisting a group towards a common goal (62%); and 2) inspiring others (23%). For the **Mid-level Leaders**, two different common themes emerged; leadership entails: (1) collaborative actions with others (50%); and (2) team building (50%). **Senior leaders** conceptualizations could be summarized in three common leadership themes: (1) alignment (50%); (2) servant leadership (33%); and (3) inspiration (17%).

**Fig. 1:**
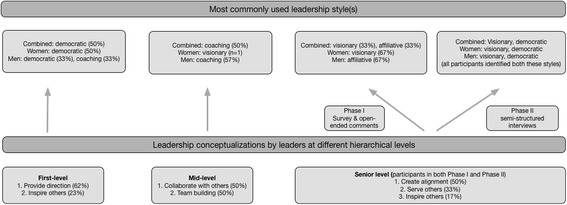
Leadership styles related to leadership conceptualizations (Phase I)

Rank order of styles and group differences (see Table 2):

The most frequently used leadership style by the first-level leaders (50%) was the democratic style, followed by coaching in both the second (43%) and third ranked (29%) positions. Women within this group identified democratic (50%) as their top ranked style, while men identified both democratic (33%) and coaching (33%) as their top leadership style. Most mid-level leaders (50%) relied on the coaching style as their first and second (38%) ranked styles, followed by affiliative as the third ranking style (38%). This pattern was reflective of the male participants in this group (*n* = 7) while the single female participant identified visionary followed by coaching and democratic as the top three leadership styles. Senior leaders did not identify one dominant style, but most commonly used visionary (33%) and affiliative (33%) styles, followed by democratic (50%) and coaching (50%) as the second and third ranked styles. Women identified visionary (67%), democratic (67%) and coaching (100%) as the top three styles in decreasing order of frequency, whereas men identified affiliative (67%) as their most frequent style, and coaching (33%), democratic (33%) and pacesetting (33%) as their second most used style. Figure 1 depicts how the leaders at different levels conceptualized leadership and their most commonly used leadership styles.

Within each leader group, Friedman tests revealed significant differences in the ranked leadership styles most commonly used for first-[χ^2^(5) =71.338, *p* < 0.001], middle- [χ^2^(5) =23.139, *p* < 0.001], and senior- [χ^2^(5) =15.788, *p* = 0.007] level leaders; as well as across gender [female = χ^2^(5) =34.311, *p* < 0.001; male = χ^2^(5) =34.92, *p* < 0.001]. Table 3a displays the rank orders for the different leadership styles within each group separately. For the first-level leaders, post hoc comparisons revealed significant differences in the ranked order of the dominant leadership style (democratic) as being ranked significantly higher than affiliative, pacesetting, and commanding styles(*p*s < 0.05). Middle-level leaders ranked their dominant leadership style (coaching) as significantly more used than pacesetting and commanding styles (*p*s < 0.05). Among senior-level leaders [χ^2^(5) =19.00, *p* = 0.002], post hoc comparisons did not reveal any significant differences between ranked leadership styles except between democratic and commanding styles (*p* < 0.05). Across all participants, the differences within gender showed that female and male participants ranked their dominant leadership style (democratic and coaching styles respectively) as significantly more used than both pacesetting and commanding styles (*ps* < 0.05).

### Phase II

#### Semi structured interviews

The following themes were identified. Table 3b displays the leadership styles used by senior-level leaders who were interviewed.

**Although most senior leaders prefer democratic and visionary styles, pace-setting and commanding styles are not uncommon:** All eight senior leaders described the use of democratic and visionary styles as the most preferred and the most commonly used styles. Most senior leaders used language to reflect democratic style such as, “my leadership style is very much built around generating consensus, bringing people along carefully and I do not tend to be the way out in front or a vocal follow me kind of a leader.” Another leader recalled, “I have used mostly the collaborative style but I have become authoritarian, when I have to.” Most senior leaders found that they had to use the pace-setting style when working with physicians as reflected in the comments, “I am a bit more directive…….most often with physicians,” and “…. who are often difficult to engage and need to be prodded towards organizational goals, but when get motivated produce high-quality results.” The leaders recalled, “to get movement with busy physicians on some issues to be addressed,” e.g., around creating policies, when “it’s really like pulling teeth,” they had to do, “some initial work themselves” and “drop in on the work” themselves to ensure its’ progress. The leaders were cognizant that this may come across as, “autocratic, because you have to get a job done and it’s really hard.”

**Even for a specific situation, as the work progresses, styles may need to change to facilitate progress**: Most senior leaders were comfortable with, “really good leaders understand the concept of situational leadership… and I have had to adapt my styles…” A common theme was that to achieve results in a timely manner leaders often had to move from visionary, through collaborative to pace-setting /directive styles as reflected in, “my job is to help enable things to happen and get out of the way of bright people who can make it happen,” however, “if you cannot reach consensus, then I change my tactics and if a decision needs to be made, then a majority decision – if that doesn’t work then I make a decision and take responsibility for it.”

**Leaders account for the influence of contextual factors and organizational needs**: All senior leaders took into account the larger context in which they were operating, as captured in phrases like, “how the college of medicine is situated in and affected by the overarching changes at the university,” “the changes at my institution are affected by the national conversation around educational reform,” and “the work involves fundamental organization-wide deep changes such as a drastic culture change.”

**Leaders consider how they themselves are situated in the organization and the situation**: One senior leader who had an “easy going” personality and preferred to develop personal relationships found himself in a leadership position where the “culture was very position authority oriented, a little bit formal, a little bit too rules-oriented” limiting the ability to “joke, say something that could be misunderstood.” Another senior leader remarked, “I need to know where I stand with people and how am I perceived?”

**Staying authentic to the true nature of “self” makes some styles difficult to practice**: According to one senior leader, “I prefer collegial, collaborative, friendly….that’s my preferred modus operandi, …..but when I needed to be autocratic and more directive than I would choose, that is intentional and hard work for me, that is not intuitive.” Another leader mentioned “where I am not doing what comes naturally and I am consuming energy to behave in ways that are not natural or intuitive for me” it creates “stress points,” “as I want to be controlling the outcome but not by overly controlling the people.”

**As the leaders mature they become more adept at using multiple styles**: Senior leaders were very frank in describing their leadership journey alluding to using a smaller repertoire of styles, more use of autocratic style - “save time, see it my way” early on in their careers to more collaborative and participative styles and expressing divergent opinions in more engaging language such as, “I actually see that differently” compared to earlier phases, “I am right and you are wrong.” However, the journey for some leaders was in the opposite direction, where early on in their careers they were collaborative to the point of being ineffective and had to learn to use “firm” styles such as pace-setting and directive to achieve results.

## Discussion

**Fig. 2:**
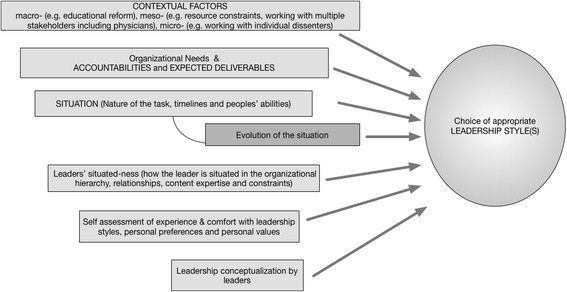
Identified factors affecting the use of leadership styles in medical education (Phase II)

Using a mixed methods approach this study on 42 participants identified differences in the repertoire and preferred styles at different levels of medical education leadership. These differences are likely to reflect participants’ conceptualization of leadership, expected accountabilities and deliverables, and adaptation to the situation and latter’s evolution, the larger context, leaders’ awareness of how they are themselves situated, and personal preferences. These are depicted in Fig. 2 and discussed below.

The top three most frequently used leadership styles across all leadership levels were democratic, coaching and visionary (authoritative) styles. These three styles are highly positive, and create resonance within the organizations with the potential to boost performance [18]. Our finding that leaders at all three levels were drawing from a large repertoire of styles is consistent with an earlier report that effective leaders are able to utilize a wide range of leadership styles [28]. However, five out of eight senior leaders (semi-structured interviews) did not use coaching or affiliative styles (discussed below).

The preferred use of the democratic style by the first-level leaders could be reflective of their main leadership conceptualization and the interactions with their peer group over whom they have limited, if any, positional authority; the democratic style provides the best opportunity for collaboration with the stakeholders feeling “being heard.” Middle -level leaders are responsible for larger cross-discipline programs and their top ranked leadership style, coaching, also fits in with this level of responsibility as well as their perception that leadership is about working with and developing others; the coaching style typically connects the goals of the followers with the organization’s goals [37] and is a good engagement strategy to promote wider ownership. The senior-level leaders frequent use of visionary and affiliative (Phase I) and visionary and democratic (Phase II) styles as their top ranked styles could indicate a) a broader conceptualization of leadership and a more robust internalization of EI, b) the necessity to engage a wider group of stakeholders over whom they have some positional authority, b) the broad spectrum of accountabilities of senior level positions, and c) their experience and maturity in leadership roles and comfort with the artful practice of leadership.

These findings highlight differences in leadership styles at different levels within the organizational hierarchy where different leadership roles fundamentally serve different purposes (e.g., team leaders with managerial accountabilities vs. strategic leaders involved in policy creation). This idea has some support in the literature; differences in leadership styles were shown between first-, middle-, and senior- leaders in diverse UK organizations, with participative (similar to democratic) and delegative (similar to visionary) leadership styles used increasingly more often with increases in leadership levels [38]. Indeed, a wider range of leadership behaviours in upper leadership positions has been shown to be associated with leader effectiveness in various industries including construction [39] and marketing [40] and other business sectors [41]. However, other factors, such as age and number of years in leadership roles, could have also contributed to differences in the use of specific leadership styles. Previous research has highlighted subtle differences in the use of leadership styles of managers and leaders in various UK organizations [42], with older managers favoring more participative styles than younger managers.

Although, generalizations are fraught with risk, and a deeper analysis of gender differences was not the aim of this study, the overlap in the use of leadership styles by men and women, especially among the first level leaders, may be linked to similar professional identities and leadership conceptualizations. Overall, the democratic style was most frequently used by women leaders whereas their male counterparts placed more emphasis on a coaching style. These findings are somewhat consistent with earlier reports, which showed that women generally make use of more democratic/participative leadership styles whereas men utilize more of a directive/autocratic leadership approach [43]. A preference by women leaders for leadership styles associated with greater effectiveness [44] has been attributed to women’s use of feelings, greater emotional intensities [45, 46], and attention to social sensitivities prior to taking action [47]. Although there are only minimal differences in the leadership ability of men and women [15], women’s leadership styles have been defined as more people-based [48] and collaborative and relationship oriented [49] and this could be leveraged in leadership development programs.

Many contextual factors, which can be considered at three different levels – macro-, meso- and micro - affected a leader’s use of different styles. The senior leaders (phase II) identified the ongoing educational reform - articulated in multiple reports including FMEC-MD [6], FMEC-PG [7], and health professionals for a new century [50] - as the key national-level contextual factor that affected their interactions with stakeholders. Visionary, democratic and coaching styles helped with this engagement through collaborative leadership efforts to guide their thinking, behavior, and outcomes.

A factor associated with the use of pace-setting styles was working with the physicians, who are often difficult to engage. This challenge is likely reflective of the inherent knowledge work nature of physicians and the attendant need for autonomy [51] and associated intrinsic conflict between professionalism and bureaucracy [52]. A surprising finding of our study is that many senior leaders used the commanding and pace-setting styles more often than was expected. It could be that the study sample reflects a higher proportion of those leaders who took charge at a time that required galvanizing change in their organization, e.g., fixing multiple ongoing accreditation issues, or changes in the identity of the organization as opposed to building upon strengths in an already established institution. These two styles often needed to deliver on challenging organizational outcomes require the apex leaders to hold people to high standards [21]. This is also a likely reason for the non-use of coaching and affiliative styles by five out of eight senior leaders in our study (phase II). The use of pace-setting and commanding styles often required reparative work on relationships after the fact or involved drawing upon the relationship capital accrued earlier. This is consistent with an earlier observation that when used too frequently these styles can create dissention and conflict within an organization [18]. A few senior leaders identified the need for supporting others prior to complex and difficult undertakings, which is consistent with the finding that leaders with a high EI provide socio-emotional support before the pressures linked to tasks come into play [53].

An important insight from our study is the senior leaders’ cognizance of how they themselves are established in the “situation” in terms of their position in the organizational hierarchy, relationships with and perceptions of the people, their own content expertise, and constraints. This highlighted the use of emotional intelligence (e.g., self-awareness, self-regulation, motivation, empathy and social skills [37]) required to adapt their style to the peoples’ preferences, motivations and willingness to engage.

## Conclusions

The findings from this study have helped elucidate how medical education leaders use different style(s) at each leadership level and the appropriateness of these styles for different accountabilities – with senior leaders using a broader range of styles. Leadership education could be broadened to include the knowledge and use of different leadership styles, especially at first- and middle- level positions. Since EI can be cultivated and refined through emotional competency training and coaching [28]; it is useful to include it in leadership development initiatives within medical education [26, 54, 55]. For the practice of leadership, the styles (i.e. preferences for interactions with people), although prescribed for different situations, need to be rooted in personal philosophy of leadership, and ones’ values and beliefs to be an authentic leader. Mere practice of superficial behaviors may not be sufficient, and often counterproductive when people sense artificiality and pretense. Our results highlight several factors (Fig. 2) affecting the use of leadership styles, which could be considered when moving nimbly between styles. A flexible repertoire of four or more styles makes a highly effective leader [28], so some styles would need to be deliberately developed through formal leadership development combined with an inner journey rooted in self-discovery.

### Limitations of the study and future investigations

The findings of our study should be considered in light of the following limitations: (1) participation was limited mostly to leaders at one institution and specific leaders at the national level thereby affecting affect transferability to other settings, (2) the study did not include analysis of objective data on leadership effectiveness, such as performance reviews of leaders or self-reflection of effectiveness, and (3) small sample sizes within leader levels and limited demographic information in the first-level leader group limit generalizability. Future investigations may explore correlations between leader behaviour, EI measures and leader effectiveness and deeper dimensions of gender differences.

## Additional file

Additional file 1: Semi-structured interviews. Interview guide to semi-structured interviews. (DOCX 93 kb)

## Abbreviations

EI: Emotional Intelligence
FMEC MD: The Future of Medical Education in Canada Medical Doctor
FMEC PG: The Future of Medical Education in Canada Postgraduate
