# Supplementary material for: Goleman’s Leadership styles at different hierarchical levels in medical education
Source: BMC Med Educ. 2017 Sep 19;17:169. doi: 10.1186/s12909-017-0995-z (PMC5606110; doi:10.1186/s12909-017-0995-z)
Supplement: Additional file 1: — Semi-structured interviews. Interview guide to semi-structured interviews. (DOCX 93 kb) [file 12909_2017_995_MOESM1_ESM.docx]

**Interview Guidelines**

- Introduction to interviewee and consent procedure.
- The purpose of the study is to develop a framework of leadership for medical education and contribute to the literature on leadership in medical education, based on an analysis of the perceptions of key health education leaders in Saskatchewan medical education organizations at the national level in Canada. Overall, we want to gain an understanding of deeper dimensions of leadership by gathering information through your personal “lived” leadership experiences.

Interview Questions

| 1 | Would you please describe your journey to the current leadership position?  What would you say are some of the key leadership skills that have served you well in your current position and how have you acquired these? |
| --- | --- |
| 2 | Could you describe a time when you consciously worked to rally your organization around a common vision in medical education? What was the vision? Did the people rally? |
| 3 | It is said that most leaders have preferred styles of leading. Could you share an example of how you have adjusted your leadership style to a particular situation? |
| 4 | As you think back to your experience with a significant organizational change, how would you describe your approach, initial steps, mid-course corrections and what was the outcome? Did you learn anything from the experience? |
| 5 | Is there anything else you would like to say that would help me think more clearly about medical education leadership and its features? |
| 6 | Thank you for your time [and describe the post-interview process]. |
